# In Vitro and In Vivo Effects of Resveratrol on Rat Hepatic CYP1A2

**DOI:** 10.3390/ph18111633

**Published:** 2025-10-29

**Authors:** Sandra Luz Hernández-Ojeda, Raquel López-Arellano, Carla O. Contreras-Ochoa, Daniel Hernandez-Patlan, Rafael Camacho-Carranza, Antonio Romo-Mancillas, Giovanny Aguilera-Durán, Carlos A. Hernández-Gallardo, Ricardo I. Cárdenas-Ávila, Tzipe S. Govezensky, Elvia A. Morales-Hipólito, Javier J. Espinosa-Aguirre

**Affiliations:** 1 Instituto de Investigaciones Biomédicas, Universidad Nacional Autónoma de México, Tercer Circuito Exterior, Ciudad Universitaria, Ciudad de México 04510, Mexico; slhernandez@iibiomedicas.unam.mx (S.L.H.-O.); rcamacho@iibiomedicas.unam.mx (R.C.-C.); carloscahg60@gmail.com (C.A.H.-G.); ricardo.cardd@ciencias.unam.mx (R.I.C.-Á.); tzipe@unam.mx (T.S.G.); 2 Laboratorio 5: Laboratorio de Ensayos de Desarrollo Farmacéutico (LEDEFAR), Facultad de Estudios Superiores Cuautitlán, Universidad Nacional Autónoma de México, Cuautitlán Izcalli 54714, Estado de México, Mexico; lopezar@unam.mx (R.L.-A.); danielpatlan@comunidad.unam.mx (D.H.-P.); eadriana_mh@comunidad.unam.mx (E.A.M.-H.); 3 Centro de Investigación Sobre Enfermedades Infecciosas, Instituto Nacional de Salud Pública, Av. Universidad no. 655, Santa María Ahuacatitlán, Cuernavaca 62100, Morelos, Mexico; ccontreras@insp.mx; 4 División de Ingeniería en Nanotecnología, Universidad Politécnica del Valle de México, Tultitlán 54910, Estado de México, Mexico; 5 Grupo de Diseño Asistido Por Computadora y Síntesis de Fármacos, Facultad de Química, Universidad Autónoma de Querétaro, Centro Universitario, Querétaro 76010, Mexico; ruben.romo@uaq.mx; 6 Laboratorio de Química Cuántica y Modelado Molecular, Unidad Académica de Ciencias Químicas, Universidad Autónoma de Zacatecas, Zacatecas 98160, Mexico; giovanny.aguilerad@uaz.edu.mx

**Keywords:** resveratrol, cytochrome P450, chemoprevention, CYP inhibition

## Abstract

**Background/Objectives:** Although several studies have been reported on the modulation of Cytochrome P450 by resveratrol, inconsistencies in the results obtained require further investigation. Here, we report the results of in vivo and in vitro experiments investigating the effect of resveratrol on CYP1A2, which participates in the biotransformation of several drugs used for the treatment of human malignancies. **Methods:** Male Wistar rats were exposed to resveratrol through diet (1%) for 30 days, and the hepatic CYP1A2 activity and protein concentration were assayed at the end of the treatment. Additionally, the capacity of the phytochemical to interfere with the induction of CYP1A2 by benzo[a]pyrene (50 mg/kg body weight) was also studied. The inhibition of CYP1A2 activity in rat liver microsomal and recombinant human enzymes by resveratrol, as well as its inhibitory kinetics and type of inhibition, were compared. **Results:** No significant increase in the protein concentration of hepatic CYP1A2 was found in resveratrol-treated rats, but it induces CYP1A2 activity and enhances the induction effect of benzo[a]pyrene. In silico and in vitro experiments demonstrated that resveratrol binds to the active site of human CYP1A2 through hydrophobic interactions with PHE125, PHE226, PHE260, and ALA317, and hydrogen bonds with SER122 and ASP313. It inhibits human recombinant CYP1A2 activity as well as that in rat liver microsomes, with IC_50_ values of 46 µM and 485 µM, respectively. Resveratrol showed a mixed type of inhibition of recombinant human protein and a competitive inhibition of rat liver microsomal CYP1A. **Conclusions:** We can conclude that resveratrol is an in vitro inhibitor of CYP1A2, but it increases the benzo[a]pyrene CYP induction effect in vivo.

## 1. Introduction

Resveratrol (trans-3,4′,5-trihydroxystilbene) (RSV) is a natural polyphenolic molecule found in the flowers, nuts, fruits, bark, and seeds of several plants, and is a fundamental part of the human diet [1]. RSV exhibits a range of biological activities and characteristics that are beneficial to human health, including anti-aging, antioxidant, anticancer, anti-inflammatory, and antifibrotic effects. RSV has been demonstrated to protect against several types of cancer, including prostate, lung, and breast cancers, although the mechanisms responsible for these chemopreventive actions are not fully understood [2,3,4]. Due to these properties, RSV has been proposed as a chemopreventive agent by reducing the incidence of carcinogenesis by intervening at one or more of its several stages [5,6]. Several clinical trials have been reported, including those involving healthy volunteers and cancer patients, in which doses ranging from 25 to 5000 mg were administered over 1 or 28 days [7].

Several types of cancer chemopreventive agents have been reported, including so-called blocking agents that act at the initiation of the carcinogenic process by inhibiting the “activation” of procarcinogens, interfering with Phase I metabolizing enzymes, or enhancing the DNA repair process or antioxidant activity [8]. The superfamily of hemoproteins known as Cytochrome P450 (CYP) catalyzes the biotransformation of procarcinogens, drugs, and endogenous substances; therefore, it has been identified as a possible target for cancer chemoprevention [9]. The chemopreventive action of RSV could result from its capacity to inhibit CYPs involved in the metabolism of environmental carcinogens, such as benzo[a]pyrene (BP) and heterocyclic amines. However, inhibition of CYP activity could result in alterations in the absorption, distribution, metabolism, and excretion (ADME) of co-administered drugs.

Chun et al. (1999) reported that RSV inhibits CYP1A1-associated ethoxyresorufin-*O*-deethylase activity (EROD) in human liver microsomes and displays potent inhibition of human recombinant CYP1A1/2, showing over 50-fold selectivity for CYP1A1 [10]. These data were confirmed by Chang et al. (2001), who provided evidence of the inhibitory properties of RSV against human recombinant CYP1B1, CYP1A1, and CYP1A2 through a mixed type of inhibition [11].

Although results from in vitro experiments suggest an inhibitory effect of RSV on the CYP1A subfamily, as far as we know, there are no human studies to corroborate this effect. On the contrary, an RSV inducing CYP1A2 activity (decreasing the caffeine/para xanthine ratio) was reported by Chow et al. (2010), in a clinical study in which participants were given 100 mg of caffeine after 1 g/day of RSV (for four weeks), indicating a potential drug interaction possibility [12]. Another study in rats reported a slight increase in CYP1A2-associated methoxyresorufin-*O*-demethylase activity (MROD), suggesting a positive modulation of CYP1A2 by RSV (100 mg/kg for one week in a semisynthetic diet); however, the relative content of CYP1A2 mRNA was reduced by 25% [13].

Here, we studied the effect of sub-chronic RSV oral consumption on the activity and protein concentration of non-induced and BP-induced rat hepatic CYP1A2, as well as the in vitro inhibition and enzyme kinetics of MROD activity in rat liver microsomes and human recombinant CYP by RSV. In silico studies of the interaction of RSV with CYP1A2 were also included.

## 2. Results

**Figure 1:**
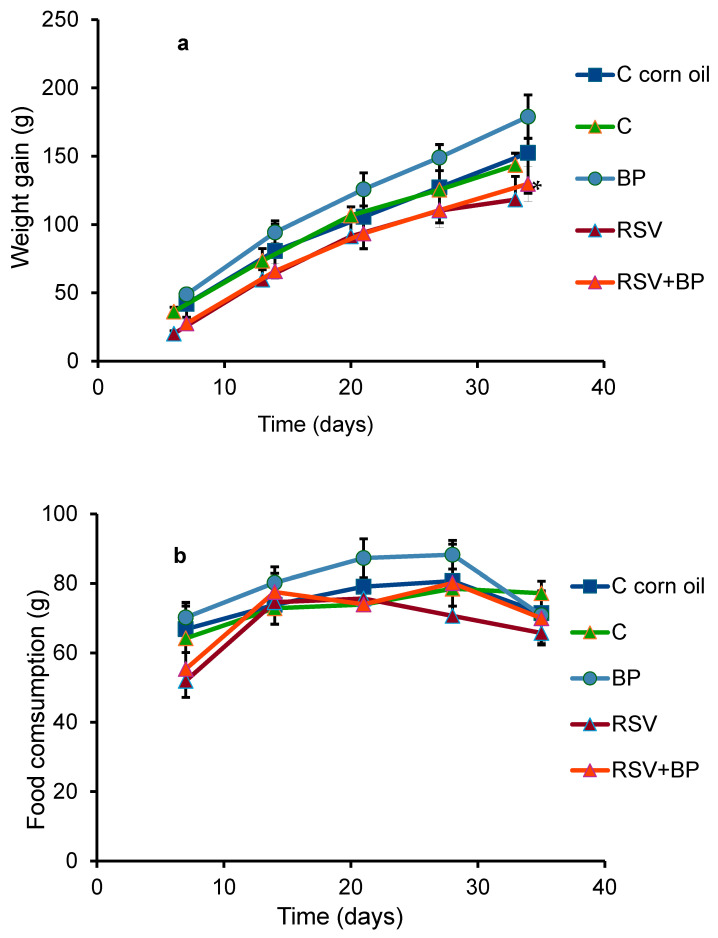
Weight gain represents the relation: body weight at the indicated time—body weight on the first day of the experiment (**a**) and food consumption (**b**) of the animals over 30 days of treatment, as described in Section 4. Data presented as mean ± SEM; * *p* ˂ 0.05. *n* = 6 in each group.

The variation in the weight gain of the animals in each group is shown in Figure 1a. The RSV-treated animals exhibited a significant difference compared to their control group; however, this difference was not evident in the body or liver weights of the animals at the end of the experiment (Table 1). No differences in behavior were noted between the groups. The pellet consumption was equal in all groups, with a slight, non-significant decrease in the group fed the RSV diet (Figure 1b). RSV plasma concentrations in each group of rats (six animals/group) ± SD at the end of the experiment are shown in Table 2. Similar concentrations were found in the groups of animals treated with RSV and RSV plus BP.

**Figure 2:**
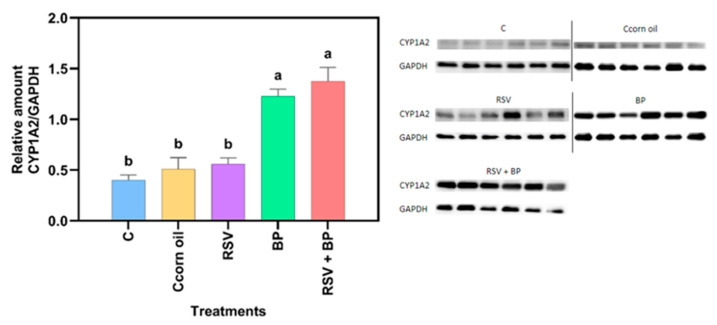
CYP1A2 protein concentration of animals over 30 days of treatment, as described in Section 4. Representative Western blot results from each of the six animals are presented. The results are expressed as mean ± SE (*n* = 6). Two independent determinations were run. Different letters above each bar mean the statistical difference between treatments (*p* < 0.05).

**Figure 3:**
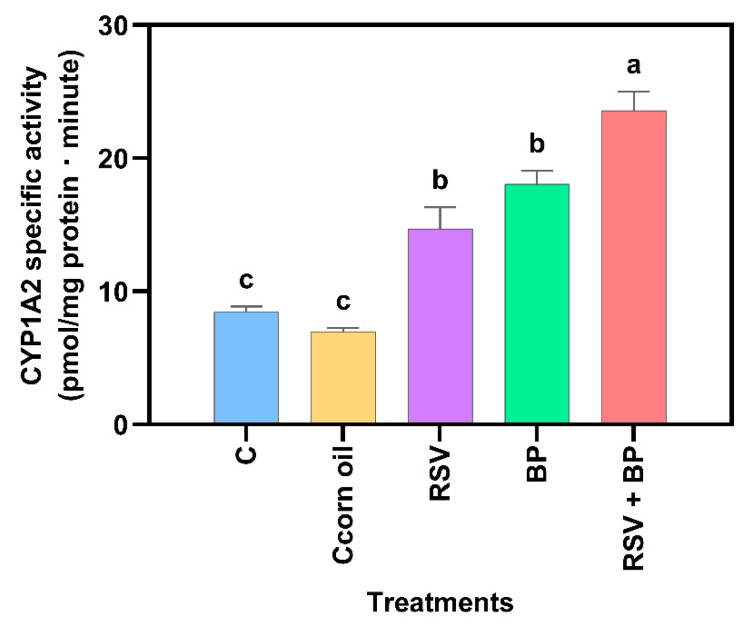
CYP1A2 liver microsomal protein specific activity of animals after 30 days of treatment, as described in Section 4. The results are expressed as mean ± SE (*n* = 6). Two independent determinations, each with three replicates/sample/determination, were performed. Different letters above each bar mean the statistical difference between treatments (*p* < 0.05).

Compared to the control group, no significant increase in protein concentration was noted in the RSV-treated animals (Figure 2). The effect of RSV on the CYP induction properties of benzo[a]pyrene (BP) is shown in Figure 2 and Figure 3. As expected, BP induced protein concentration and CYP activity, while RSV potentiated BP activity but not protein concentration, suggesting a posttranscriptional mechanism of RSV, although this needs to be experimentally proven.

**Figure 4:**
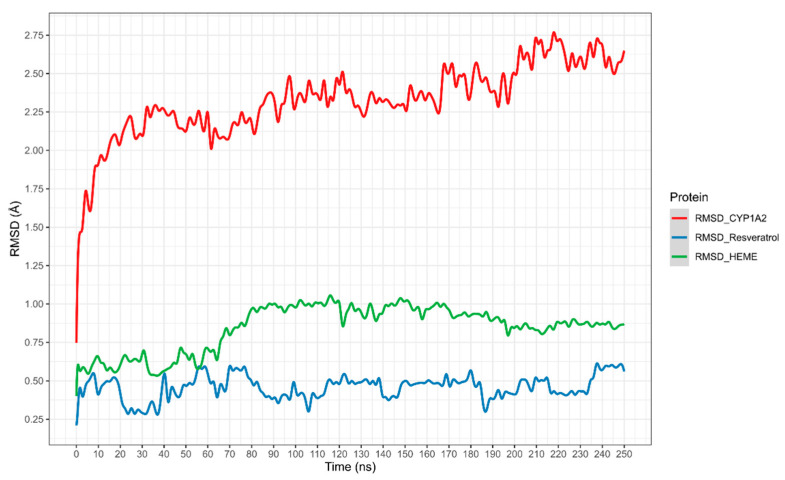
Root mean square deviation (RMSD) average of the CYP1A2-RSV complex over time.

The potential interaction of RSV with CYP1A2 was first assessed using in silico experiments. The results, shown in Figure 4, display the root mean square deviation (RMSD) of the CYP1A2–RSV complex over simulation time, calculated as the average of three independent simulations (the RMSD of three independent simulations is present in Supplementary Information Figure S1). The complex exhibited a slight shift in CYP1A2 movement between 2.25 and 2.75 Å. The heme group exhibited higher mobility regarding RSV, which stabilized from 80 ns to 250 ns. Meanwhile, RSV stabilized from 90 ns, with a slight decrease in movement between 190 and 230 ns.

**Figure 5:**
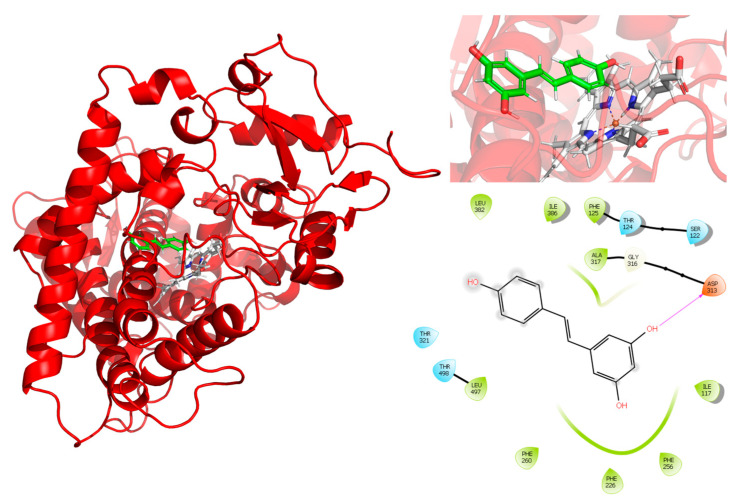
Ligand interactions between resveratrol (green) with residues in the active site of CYP1A2 and the heme group (gray). Hydrophobic interactions (green), polar interactions (pale blue), and hydrogen bonds (red).

For the interaction analysis, the structure obtained from the clustering of the concatenated trajectories was selected. Interactions between RSV and the active site of CYP1A2, shown in Figure 5, exhibit a hydrogen bond interaction between RSV and Asp313. The interactions observed in the PLIP server are hydrophobic interactions with PHE125, PHE226, PHE260, and ALA317, and hydrogen bonds with SER122 and ASP313, which indicate the relevance of the interactions between the aromatic rings of RSV and the protein.

Additionally, metadynamic simulations were performed in triplicate to estimate the theoretical binding energy (ΔG) of RSV with CYP1A2, with an average ΔG of −94.13 kcal/mol. The ΔG and observations from the RMSD plots suggest that RSV forms a stable complex with CYP1A2.

**Figure 6:**
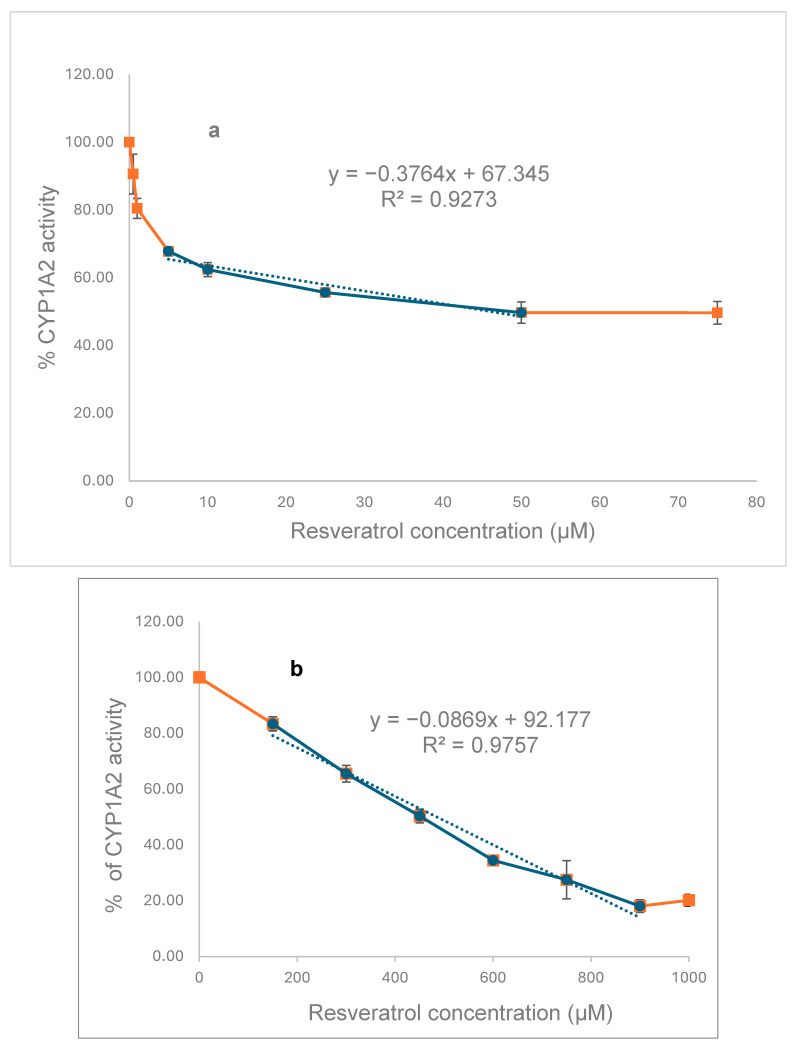
Inhibition of MROD activities of recombinant CYP (**a**) and that of rat liver microsomes (**b**) by resveratrol. The mean of two independent experiments ± SD is represented at each point. IC_50_s of 46 µM and 485 µM for human and rat CYP, respectively, were calculated from the linear portion of the line.

To explore the in vitro effects of RSV on CYP1A2, we initially investigated the inhibition of MROD activities in recombinant CYP (Figure 6a) and rat liver microsomes (Figure 6b). RSV inhibits the CYP1A2 activity of both recombinant protein (IC_50_ = 46 µM) and that present in liver microsomes (IC_50_ = 485 µM), with the human protein being ten times more sensitive to RSV than that present in rat liver.

**Figure 7:**
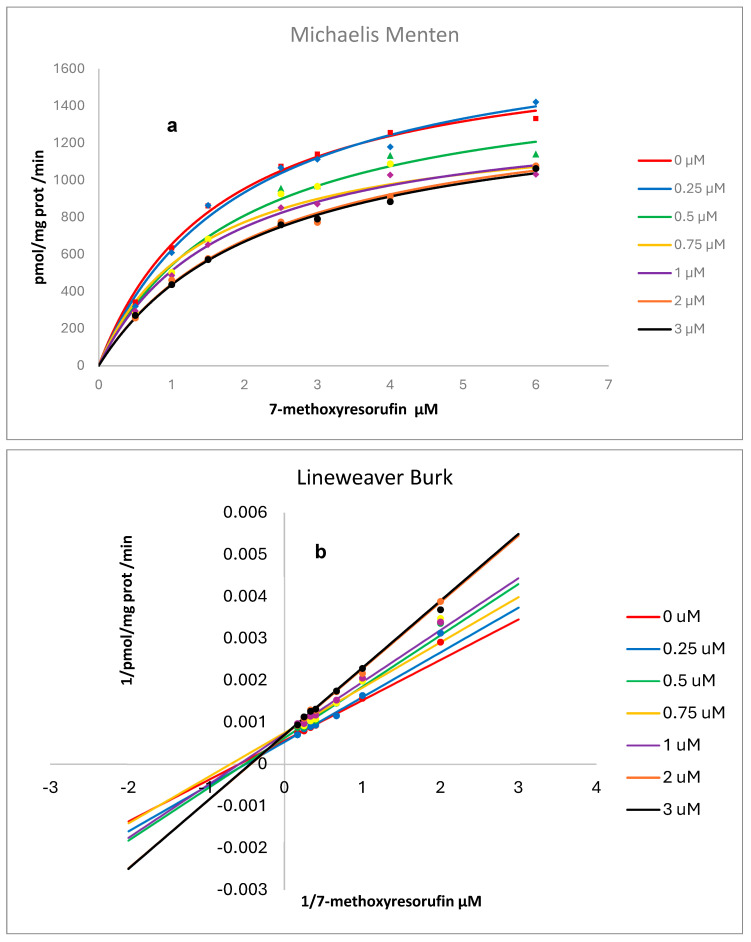
Effect of resveratrol on human recombinant CYP1A2 (MROD) activity. Plots of velocity versus substrate concentration. Each point represents the mean obtained from three independent experiments ± SD (three replicates/experiment) (**a**) and Lineweaver–Burk plots (**b**). The best fit, as determined by GraphPad Prism software, is represented by solid lines.

**Figure 8:**
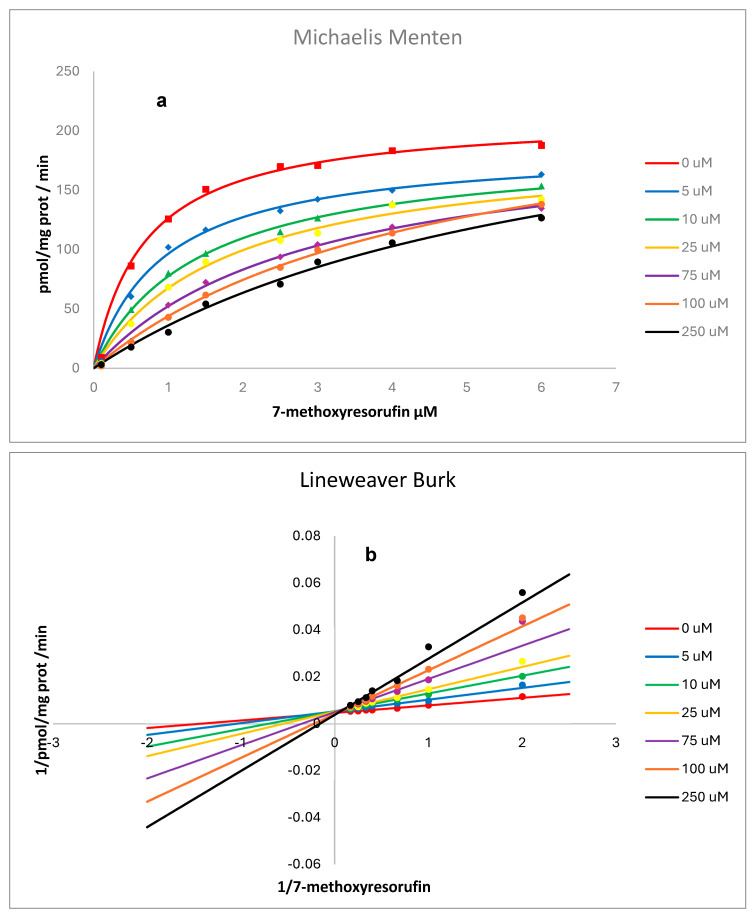
Effect of resveratrol on rat liver microsome CYP1A2 (MROD) activity. Plots of velocity versus substrate concentration. Each point represents the mean obtained from three independent experiments ± SD (three replicates/experiment) (**a**) and Lineweaver–Burk plots (**b**). The best fit, as determined by GraphPad Prism software, is represented by solid lines.

Kinetic analysis resulting from the presence of various concentrations of RSV on the activity of human recombinant and rat liver microsomes, CYP1A2, is shown in Figure 7 and Figure 8. Non-linear regression analysis and Lineweaver–Burk plots pointed to a mixed-type inhibition for RSV over human CYP and a competitive inhibition over rat liver microsomes with values for Ki (CI 1.906 to 19.17), Km (CI 1.217 to 2.200), and Vmax (CI 1532 to 1887) of 4.03 µM, 1.67 µM, and 1691 pmol/mg protein/min., respectively, for human CYP and Ki (CI 31.52 to 76.41), Km (CI 0.6279 to 1.308), and Vmax (CI 176.7 to 214.9) of 49.1 µM, 0.918 µM, and 194 pmol/mg protein/min., respectively, for CYP in rat liver microsomes [14]. Goodness-of-fit metrics are provided as Supplementary Information (Table S1).

## 3. Discussion

RSV is a constituent of human diets; it is mainly found in grape skin, mulberries, and peanuts. RSV is an antimicrobial phytoalexin produced by plants in response to the aggression of microorganisms and other abiotic stresses [15].

Due to its several beneficial biological properties, RSV has been proposed as a cancer chemopreventive agent [16]. In vitro studies have provided substantial evidence of its anticancer potential against various cell lines, including those from the liver, breast, prostate, lung, pancreas, and colon [17]. Animal in vivo studies involving xenograft and murine models support the findings mentioned above, highlighting the beneficial effects of RSV in reducing oxidative stress, improving cardiovascular health, and inhibiting tumor growth [17]. Based on the results mentioned earlier, several human clinical trials were conducted, including the administration of oral supplements with RSV doses ranging from 25 to 5000 mg/day for durations of 1 to 28 days. RSV maximal plasma concentrations vary from 1.48 to 1942 ng/mL depending on the dose and time of treatment. These clinical trials included healthy volunteers and patients with colorectal and hepatic cancer [7]. In our study, the mean RSV rat plasma concentration at the end of treatment was 829 ng/mL (Table 2) in animals consuming 1% RSV, which falls within the range of human concentrations reported in clinical trials. Considering the food ingestion and weight variation during the experiment, we estimated that the RSV dose was 2.5 g/kg/day.

Taking into account that no data were available regarding the possible effect of RSV on CYP modulation at these higher concentrations, we chose 1% RSV-supplemented nutritional pellets for this study bearing in mind that inhibition or induction of Phase I enzymes by natural or food compounds can modify the ADME process of the co-administered drugs, leading to alteration in drug plasma levels and its toxicity [18]. Particularly, CYP1A2 is a hepatic enzyme that metabolizes a range of drugs, including clozapine, cyclobenzaprine, imipramine, mexiletine, and theophylline. It is responsible for the biotransformation of 10% to 15% of drugs undergoing metabolism [19]. It is also involved in the biotransformation of carcinogenic heterocyclic aromatic amines resulting from meat cooked at high temperatures.

Although the RSV-treated group showed a reduction in body weight gain compared to controls (Figure 1a), the ratio of liver weight/body weight of the animals at the end of the experiment did not show differences between the groups (Table 2). Additionally, no differences in food consumption (Figure 1b) or behavior were seen in the group of animals treated with RSV. Our data partially agree with Chiba et al. (2016), who reported that C57BL/6J male mice were nourished with diets including 0.005%, 0.05%, or 0.5% (*w*/*w*) RSV for 1 or 12 weeks [20]. None of the RSV doses affected body weight in the group of mice fed diets for 1 week.

Although no differences in protein concentration with respect to control were noted in the group of animals fed with food pellets containing RSV, CYP activity increased as a result of RSV exposure (Figure 2 and Figure 3). Trusov et al. (2010) observed a 42% increase in the activity of MROD in rat liver after receiving a diet with RSV (100 mg/kg) for one week [13]. However, mRNA for liver CYP1A2 reduced by 25%, suggesting a post-transcriptional mechanism exerted by RSV [13]. Casper et al. (1999) [21] demonstrated in ex vivo and in vitro models that RSV can promote AhR nuclear translocation and binding to XRE elements in the DNA; however, successive transactivation does not occur, resulting in no activation of genes under the control of the AhR pathway, including those of the CYP1 family.

The CYP1A2 induction property of RSV has also been reported in humans. An intervention study in 42 healthy volunteers exposed to 1 g/day of RSV for four weeks resulted in a 16% decrease in the caffeine/paraxanthine metabolic ratio, suggesting an induction of CYP1A2 activity (Chow, HHS et al., 2010) [12].

On the other hand, it is well known that BP induces CYP through the activation of the AhR pathway [22]. In this work, BP induced more than twice the basal activity of CYP1A2. Pretreatment of rats for 30 days with a diet containing RSV potentiated CYP1A2 activity by 38%, as induced by a single i.p. injection of 50 mg/kg BP, but the protein concentration was not different from that of the group treated with BP alone (Figure 3).

As far as we know, there is no previous report of the potentiated effect of RSV and BP on the in vivo induction of CYP1A2 activity reported here; however, our previously reported work on various flavonoids showed that 3-hydroxiflavone, 5-hydroxiflavone, quercetin, morin, kaempferol, and myricetin exhibit contradictory effects on human CYP1A1 activity, stimulating it at lower concentrations and inhibiting it at higher ones [23]. This same double action of flavonoids on human benzyloxyresorufin-*O*-debenzylase activity was observed by Siess et al. (1995), but no mechanism of action has been proposed [24]. The concentration of RSV in the rat serum was 829 ng/mL (3.4 µM), which is ten times lower than its inhibitory IC_50_ (485 µM) (Figure 6). Then, a double effect like that seen with flavonoids could be possible, which may explain, in part, the potentiation in BP activity seen in RSV plus BP-treated rats.

We cannot rule out physiological confounders resulting from the weight gain reduction observed in the RSV-treated group, which could account for its CYP1A2-induced activity alone or in combination with BP. Nevertheless, no modulation of CYP1A2 activity related to weight loss alone has been reported. Additional experiments should be conducted to clarify the mechanism behind RSV’s induction properties.

RSV inhibits EROD activity catalyzed by human recombinant CYP1A2 by a mixed type of inhibition [11]. Our results, using MROD as a substrate, also indicate a mixed type of inhibition for human recombinant CYP1A2 (Figure 7) and competitive inhibition for rat liver microsomes (Figure 8). As previously reported by us for CYP1A1, interspecies differences in CYP metabolism may account for variations in biological effects, including enzymatic activities and mutational spectra, which must be considered to improve the extrapolation from animal models to humans. Our results from in vitro experiments indicated a higher susceptibility of humans to CYP1A2 inhibition by RSV compared to the animal model.

To the best of our knowledge, this is the first time that an RSV-CYP1A2 docking simulation, including molecular dynamics, has been presented. Assuming that the complex structure of two molecules is used as the starting point, a complex is dissociated by performing a standard metadynamic run with a user-defined distance between the two molecules used as the collective variable (CV). A Gaussian energy impulse centered on the running value of the CV is periodically added to the total potential energy of the system at every time interval along the progression of the simulation, which forces the CV to increase, leading to the dissociation of the molecules [25]. Observations from the RMSD plots and the theoretical binding energy of RSV with CYP1A2, resulting from the metadynamic simulations (Figure 4 and Figure 5), suggest the high stability of the complex and a competitive inhibition profile of RSV.

The in silico data mentioned above are in concordance with the results of in vitro CYP1A2 inhibition by RSV. The predicted complex formation between CYP1A2 and RSV explains the inhibition of MROD biotransformation by human recombinant or rat liver microsome CYP1A2, with IC_50_ values of 46 µM and 485 µM, respectively. Chun et al. (1999) reported the inhibitory effect of RSV on *E. coli* membranes expressing human CYP1A2 with an IC_50_ value of 580 µM for MROD [10], which is ten times higher than that reported by us. Differences in the preparation of the reaction mixture used by them, which contains dicoumarol, may partially explain this inconsistency. In vivo treatment of rats with dicoumarol induces hepatic CYP1A2 protein and activity; therefore, dicoumarol may act as both an inducer and a substrate for CYP1A2, potentially competing with other substrates, such as MROD [26].

We can conclude that RSV inhibits CYP1A2 in human and rat liver microsomes in vitro; human CYP is ten times more susceptible to inhibition than the CYP contained in rat liver. On the other hand, exposure of rats to RSV in a sub-chronic manner, reaching serum concentrations similar to those found in human clinical trials, leads to an increase in CYP1A2 activity by itself and potentiates BP CYP induction, which may increase the toxicity of polycyclic aromatic hydrocarbons or interfere with the pharmacokinetics of drugs metabolized by CYP1A2. Additional studies are required to assess the potential RSV–xenobiotic interactions in subjects exposed to high RSV dose treatments.

## 4. Materials and Methods

### 4.1. Chemicals

Resveratrol 98% purity (Alfadelta S.A. de C.V., Naucalpan, Estado de México, Mexico); BP, corn oil, 7-methoxyresorufin, resorufin, NADPH, EDTA, DTT (Sigma Aldrich, St. Louis, MO, USA); DMSO (Merck, Darmstadt, Germany); Tris-Base (Bio-Rad Laboratories, Hercules, CA, USA); KCl, NaCl (Avantor Performance Materials, Estado de México, Mexico); glycerol (Invitrogen BioServices, ThermoFisher, Bengaluru, India). Supplies for preparing bacterial culture media were purchased from Becton Dickinson (Cuautitlán Izcalli, Estado de México, Mexico).

### 4.2. Animals and Housing Conditions

Thirty male Wistar (Rattus norvegicus) rats (weighing 180–200 g) were housed in polypropylene cages under a 12 h light/dark cycle, 50% humidity, and a temperature of 21 °C, with ad libitum access to food and water. All experimental procedures were approved and conducted in accordance with the institutional committee for the care and use of animals in biomedical experimentation (Instituto de Investigaciones Biomédicas, Universidad Nacional Autónoma de México), ID number 10385, approved on 15 April 2023. The rats used were randomly selected from different litters, and a randomization function in Excel was used to distribute them.

### 4.3. Animal Treatment

The rats were randomly assigned to one of the following five experimental groups (6 rats per group/3 rats per cage) for 30 days after one week of acclimatization: (a) non-supplemented food (5001-Laboratory Rodent Diet), (b) non-supplemented food with corn oil administration, (c) resveratrol-supplemented food, (d) non-supplemented food with i.p. administration of BP, and (e) resveratrol-supplemented food with i.p. administration of BP.

Resveratrol-supplemented nutritional pellets (1% RSV) were provided by Dr. Raquel López Arellano, Facultad de Estudios Superiores Cuautitlán, Campus 4, Universidad Nacional Autónoma de México [27]. Four days before treatment initiation and throughout the 30 days of RSV administration, weight and food consumption were registered. On the last day, animals in treatments (d) and (e) received an intraperitoneal injection of 50 mg/kg BP in 200 μL corn oil, while animals in treatment (b) received only 200 μL of corn oil. The animals were euthanized by decapitation, and their livers were collected and stored. Blood samples were collected in heparinized tubes, mixed, and centrifuged for 10 min at 190× *g*. The supernatants were stored at −80 °C. None of the animals exhibited visual signs of deleterious health; therefore, no animals were excluded, no data changes occurred during the experiment, and no adjustments were required.

Additionally, there were no other differences other than those reported. The treatments and their order were controlled by graphic descriptions of the rats’ containing boxes, as well as numbers marked on the tail with indelible ink. S.L. Hernandez-Ojeda was responsible for preserving the identity of the groups. The animal’s weight, food and water consumption, and regular activity were monitored. The experimental work was conducted in the Biological Models Unit of the Biomedical Research Institute under controlled conditions of lighting, temperature, and humidity.

### 4.4. Preparation of Plasma Samples for RSV Quantification

In total, 200 μL of plasma was mixed with 200 μL of HPLC-grade methanol (99.8%, JT Baker, Philipsburg, NJ, USA) in Eppendorf tubes and stirred vigorously for 1 min. Subsequently, 500 µL of a chloroform:methanol (50:50) mixture was added to the tubes and stirred again for 1 min. The tubes were then centrifuged at 13,000 rpm at 4 °C for 10 min, and the supernatants were transferred to new tubes for evaporation at 37 °C in a TurboVap LV evaporator with an air inlet system at a pressure of 5 and 10 pounds for approximately 30 min. After complete evaporation, the residue was reconstituted with 1 mL of mobile phase (acetonitrile: 1% formic acid, 55:45) and subsequently stirred for 1 min. Finally, the tubes were centrifuged at 13,000 rpm and 4 °C for 10 min, and the supernatant was transferred into glass vials with smaller volume inserts (200 μL) for chromatographic analysis [28].

### 4.5. Analytical Method for Determining Resveratrol

Resveratrol quantification was performed using ultra-performance liquid chromatography (UPLC) with an Acquity H-Class system, equipped with a quaternary pump, autosampler injector, and PDA photodiode array detector. For this, an ACQUITY UPLC^®^ BEH Shield^TM^ RP18 precolumn (5 mm × 2.1 mm, 1.7 μm; Waters Corporation, Milford, MA, USA) was used together with a BEH Shield^TM^ RP18 column (100 mm × 2.1 mm, 1.7 μm; Waters Corporation, Milford, MA, USA). A mobile phase composed of a mixture of acetonitrile: 1% formic acid (55:45) was used at an isocratic flow rate of 0.3 mL/min. The sample injection volume was 10 μL with an analysis time of 6 min. Empower 3 software (Waters, 2010, Milford, MA, USA) was used for data acquisition and processing. The analytical method was validated according to the International Conference on Harmonization (ICH) Q2 (R1) guideline “Validation of analytical procedures: text and methodology” [1], as well as the Food and Drug Administration (FDA) guidelines for industry, “Validation of analytical procedures and methods for drug products and biological products” [2]. The method demonstrated linearity in concentrations ranging from 1 to 15 µg/mL [29].

### 4.6. Liver Microsomal Preparation

Liver S9 fractions were prepared according to the method of Maron and Ames (1983) [30]. Briefly, the livers were minced into small pieces and homogenized in 20 mM Tris-Base, 150 mM KCl, and 1 mM EDTA (3 mL/g liver wet weight). After centrifugation at 9000× *g* for 10 min, the supernatants (S9 fraction) were recovered and then centrifuged at 100,000× *g* for 60 min. The pellet was resuspended in an equal volume of 100 mM potassium phosphate buffer (pH 7.4) and 250 mM sucrose, and the process was repeated under the same conditions. The microsomal fractions were finally resuspended in 100 mM potassium phosphate (pH 7.4), 1.0 mM EDTA, 0.1 mM DTT, and 20% (*v*/*v*) glycerol and stored at −70 °C. All the solutions and glassware were kept at 4 °C. The protein concentrations in the microsomal fractions were determined using the Bradford method (1976) [31], and the samples were then divided into 200 μL aliquots and stored at −70 °C until use.

### 4.7. Bacterial Membranes Containing Human CYP1A2

*Escherichia coli* DH5α cells expressing human CYP1A2 and NADPH P450 reductase, codified in the pCW plasmid [32] (kindly donated by Dr. Peter Guengerich, Vanderbilt University, Nashville, TN, USA), were cultured in Luria–Bertani medium supplemented with ampicillin (200 µg/mL) at 37 °C. Human CYP1A2 was expressed following the Parikh et al. (1997a) method by diluting cell cultures 1:100 in Terrific Broth/ampicillin medium (100 μg/mL) supplemented with 1 mM isopropyl ß-D thiogalactoside, 0.5 mM aminolevulinic acid, 1 mM thiamine, and trace salt [32]. Bacterial membranes were obtained according to the procedure described by Parikh et al. (1997) and preserved in potassium phosphate buffer (0.067 M, pH 7.4) containing 6 mM magnesium acetate, 20% *v*/*v* glycerol, 10 mM 2-mercaptoethanol, and a 1X protease inhibitor cocktail at −80 °C until use [32]. The total protein content was estimated using the Bradford assay.

### 4.8. Protein Immunodetection by Western Blot

In total, 20 μg of total protein was separated using 11% SDS-PAGE and transferred to 0.45 µm nitrocellulose sheets. The nitrocellulose membranes were blocked overnight with 5% albumin in TBS-Tween at 4 °C. Thereafter, the membranes were incubated overnight at 4 °C with the corresponding primary antibodies: anti-CYP1A2 (Santa Cruz Biotechnology, Dallas, TX, USA) at a dilution of 1:1000 and anti-GAPDH (GeneTex, Irvine, CA, USA) at a dilution of 1:5000. The membranes were then washed three times with TBS-Tween. The corresponding secondary antibody (anti-mouse, InvitroGen, Thermo Fisher, Waltham, MA, USA) was incubated for 1 h at room temperature (1:5000), and the membranes were then washed twice with TBS-Tween and once with TBS. The chemiluminescence reaction was performed using ECL prime Western Blotting detection reagent (Merck, Darmstadt, Germany), and the resulting images were captured with a Kodak GEL Logic 1500 imaging system (Rochester, NY, USA). Relative quantification was performed by determining band intensities using ImageJ software v1.8.0_345. Protein levels were determined from two independent experiments (*n* = 2).

### 4.9. CYP1A2 Activity

The ability of Resveratrol to inhibit CYP1A2 activity was determined by the methoxyresorufin-*O*-demethylase (MROD) assay [33]. The incubation mixture was prepared in a 96-well microplate by adding buffer (50 mM Tris-HCl, 25 mM MgCl_2_, pH 7.6), substrate (methoxyresorufin 12.5 µM in DMSO), hepatic microsomal protein (150 µg/well) or human recombinant CYP1A2 (100 µg/well), and resveratrol dissolved in DMSO at the desired concentrations. An incubation mixture with the vehicle DMSO was evaluated as a control (100% CYP1A2 activity). The final concentration of DMSO in the incubation mixture was 5%. This concentration of DMSO alone caused 13% inhibition. The microplate was incubated at 37 °C for 3 min. Then, the reaction was initiated by adding 2.5 mM NADPH. Fluorescence measurements were performed every 20 s for 40 min at excitation and emission wavelengths of 530 nm and 585 nm, respectively. CYP1A2 activity was calculated with a standard curve of resorufin (5–500 pmol/mL), and the results were expressed as pmol/mg prot/min or as a percentage of the activity of the control. The percentage of AROD activity was calculated relative to that of the vehicle (DMSO), which was assumed to be 100%. The resveratrol concentration that produced 50% inhibition in AROD activity (IC_50_) was estimated by linear regression of the linear portion of the line.

### 4.10. Kinetics of Human CYP1A2 Inhibition

Human CYP1A2 activity was assayed as previously described (Burke et al., 1994) using different concentrations of 7-methoxyresorufin (0.5–6 μM) as substrate in the absence or presence of resveratrol, 0.25–3 µM in recombinant human CYP1A2, and 5–250 µM in hepatic microsomal protein [33]. An incubation volume of 200 μL containing human CYP1A2 bacterial membranes (100 μg/well) or hepatic microsomal protein (150 µg/well) and 2.5 mM NADPH in buffer pH 7.6 (50 mM Tris–HCl and 25 mM MgCl_2_) was employed to determine the inhibition mechanism.

### 4.11. In Silico Binding Calculations

The 3D structure of human CYP1A2 (PBD ID: 2HI4, Sansen et al., 2007) co-crystalized with the inhibitor α-naphthoflavone was retrieved from the Protein Data Bank [34,35]. The protein structure was prepared using the PyMOL Molecular Graphics System (version 2.5.7, Schrodinger, LLC., New York, NY, USA), removing counter-ions, crystallographic waters, and other ligands (except the heme group). AutoDock4 (Morris et al., 2009) [36] was used to add atomic charges and solvation parameters. Molecular docking, centered on the catalytic site of human CYP1A2, was conducted using AutoDock Vina version 1.2.0 [37] and resveratrol as a ligand (ChemSpider ID392875, Royal Society of Chemistry). Binding conformation analysis was illustrated by PyMOL and Maestro (version 13.7, Schrödinger, LLC., New York, NY, USA).

To assess the stability of the binding mode calculated by docking, molecular dynamics simulations were performed with GROMACS 2023.5 [38]. Using the Solution Builder module on the web server CHARMM-GUI, a TIP3 water model solvated system was constructed with added Na+ and Cl− ions to achieve a concentration of 0.15 M and neutralize the system, incorporating the necessary parameters for simulation on the CHARMM36 force field. The system was subjected to a series of minimization and equilibration steps in an isothermal–isobaric (NPT) ensemble at 1 atm and 310.15 K. For the final production trajectory simulation of 250 ns, the simulations were performed in triplicate.

For the analysis, the representative structure (C1) of the concatenated trajectory (750 ns total trajectory) was obtained with a gmx cluster using the GROMOS clustering method. Additionally, the root mean standard deviation (RMSD) average from the relevant components of the system (Cα-protein, ligand, and hem group) was calculated using gmx rms. The plots were drawn using a ggplot in R [R Team Development, ver. 2.4.0].

The relevant protein–ligand interactions using the starting and C1 conformations were calculated using the PLIP (Protein-Ligand Interaction Profiler) [39,40] web server and the Ligand Interaction Diagram module in Maestro.

Finally, using the C1 conformation obtained from each 250 ns simulation, metadynamic simulations were performed. The systems were built in the System Builder module in the Desmond molecular dynamics module available in the academic version of the Schrödinger-Maestro 2021.4 program, with the OPLS-2005 force field, incorporating a 0.15 M NaCl concentration to neutralize the system and employing the TIP3 water model in a periodic orthorhombic box of size 20 × 20 × 20 Å. A 50 ns simulation was performed in the Metadynamics module using the collective variables: the distance between the center of mass of the proximity to residues at 5 Å of RSV and CYP1A2, and the ligands’ center of mass. Gaussian potentials were applied with a height of 0.03 kcal/mol and a width of 0.05 kcal/mol, with a restraining wall set at 20 Å [41,42].

Simulations were run in an NPT ensemble at 310.15 K and 1.01325 bar. Trajectory analysis was carried out using the Metadynamics Analysis module [43,44,45]. The values of ΔG obtained from metadynamic simulations can be interpreted as theoretical estimates of the binding free energy of RSV to CYP1A2.

### 4.12. Statistical Analysis

CPY1A2 specific activity (pmol/mg protein/min) and the relative amount of CYP1A2/GAPDH were subjected to analysis of variance (ANOVA) followed by a Tukey’s post hoc test to assess differences between treatments (*p* < 0.05). These analyses were performed using GraphPad Prism version 10.5.0 (GraphPad Software, San Diego, CA, USA). The results were reported as mean ± standard error (SE). The data for each treatment met the assumptions of normality (Shapiro–Wilk test) and homoscedasticity (Levene test).

## Figures and Tables

**Table 1:** Liver weights and liver weight to body weight ratios for rats at the end of the experiment.

Group	Body Weight (g)	Liver Weight (g)	Liver Weight/Body Weight (g)
Control	340.2 ± 20.26	12.84 ± 0.8879	0.0377 ± 0.0016
C corn oil	354.7 ± 33.43	13.82 ± 1.6429	0.0389 ± 0.0015
RSV	314.6 ± 25.01	12.57 ± 1.4968	0.0399 ± 0.0021
BP	382.3 ± 34.72	15.21 ± 1.7860	0.0398 ± 0.0037
RSV + BP	334.9 ± 25.24	13.87 ± 1.8727	0.0413 ± 0.0035

Numbers represent the mean ± SD (*n* = 6). RSV: resveratrol; BP: benzo[a]pyrene; RSV + BP: resveratrol + benzo[a]pyrene. Statistical analysis using one-way ANOVA indicated non-significant differences between the groups (*p* > 0.05).

**Table 2:** Plasma resveratrol concentration in animal groups.

Group	Resveratrol (µg/mL)	Mean ± SD
RSV	0.800	0.829 ± 0.118
	0.821	
	0.867	
	1.012	
	0.827	
	0.646	
RSV + BP	1.170	0.776 ± 0.337
	0.530	
	0.365	
	1.184	
	0.756	
	0.648	

RSV: resveratrol; RSV + BP: resveratrol + benzo[a]pyrene. No resveratrol was detected in the control, C corn oil, and BP treatments. Numbers are the results obtained from a single determination with one replicate per sample for each of the six animals per group. Statistical analysis using Student’s *t*-test with Welch correction indicated non-significant differences between RSV and RSV + BP groups (*p* > 0.05).

## Data Availability

The original contributions presented in this study are included in the article. For further inquiries, please contact the corresponding authors.

## Supplementary Materials

The following supporting information can be downloaded at https://www.mdpi.com/article/10.3390/ph18111633/s1, Figure S1. RMSD of the triplicate simulations. (A) MD1, (B) MD2, and (C) MD3; Table S1. Goodness of fit metrics regarding human recombinant and rat liver microsomes CYP1A2.
