# Supplementary material for: In Vitro and In Vivo Effects of Resveratrol on Rat Hepatic CYP1A2
Source: Pharmaceuticals (Basel). 2025 Oct 29;18(11):1633. doi: 10.3390/ph18111633 (PMC12655445; doi:10.3390/ph18111633)
Supplement: Supplementary file 1 [file pharmaceuticals-18-01633-s001.zip › pharmaceuticals-3874380-supplementary.pdf]

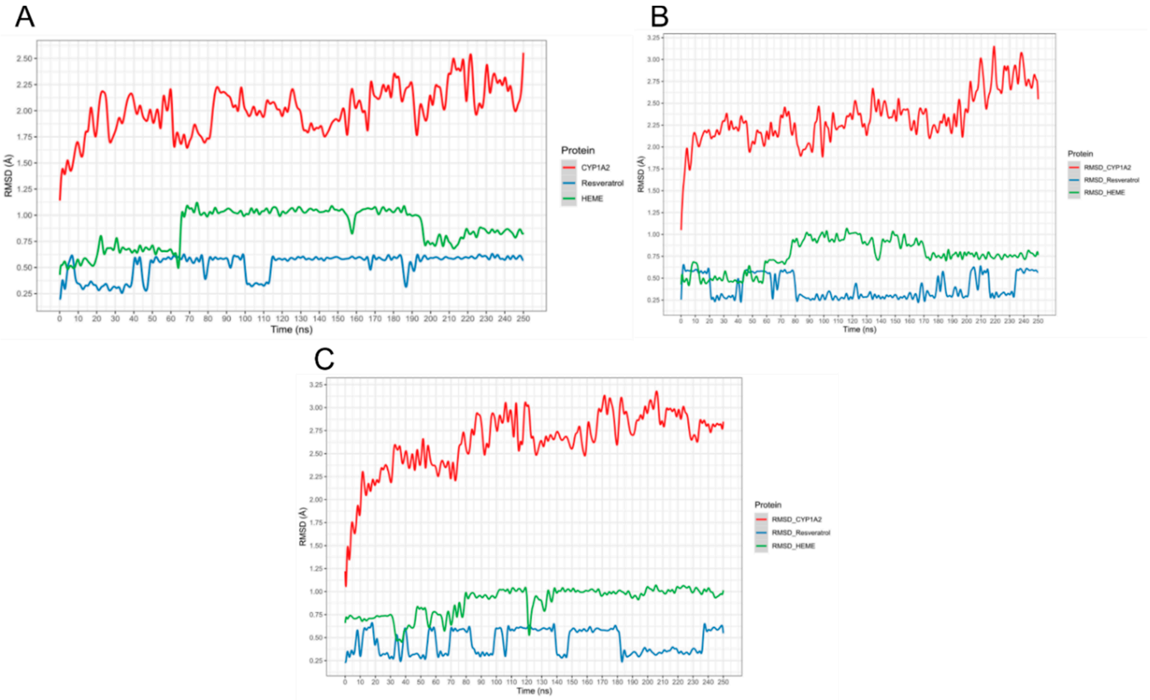

Figure S1. RMSD of the triplicate simulations. A) MD1, B) MD2, and C) MD3.

Tables S1. Goodness of fit metrics regarding human recombinant and rat liver microsomes CYP1A2.

**Mixed model inhibition**	
**Best-fit values**	
Vmax	1691
Ki	4.035
Km	1.675
**95 % CI (profile likelihod)**	
Vmax	1532 to 1887
Ki	1.906 to 19.17
Km	1.267 to 2.200
**Goodness of Fit**	
Degrees of Freedom	45
R squared	0.9507
Sum of Squares	226701
Sy.x	70.98

Goodness of fit metrics regarding recombinant CYP1A2 kinetics.

**Competitive inhibition**	
**Best-fit values**	
Vmax	194
Ki	49.15
Km	0.9187
**95 % CI (profile likelihod)**	
Vmax	176.7 to 214.9
Ki	31.52 to 76.41
Km	0.6279 to 1.308
**Goodness of Fit**	
Degrees of Freedom	46
R squared	0.8967
Sum of Squares	9702
Sy.x	14.52

Goodness of fit metrics regarding rat liver microsomal CYP1A2 kinetics.
